# Simulations of Glomerular Shear and Hoop Stresses in Diabetes, Hypertension, and Reduced Renal Mass using a Network Model of a Rat Glomerulus

**DOI:** 10.14814/phy2.14577

**Published:** 2020-09-19

**Authors:** Owen Richfield, Ricardo Cortez, L. Gabriel Navar

**Affiliations:** ^1^ Bioinnovation PhD Program Tulane University New Orleans LA USA; ^2^ Department of Physiology Tulane School of Medicine New Orleans LA USA; ^3^ Department of Mathematics Tulane University New Orleans LA USA

**Keywords:** fluid dynamics, glomerulus, mathematical modeling, renal hemodynamics, shear stress

## Abstract

A novel anatomically accurate model of rat glomerular filtration is used to quantify shear stresses on the glomerular capillary endothelium and hoop stresses on the glomerular capillary walls. Plasma, erythrocyte volume, and plasma protein mass are distributed at network nodes using pressure differentials calculated taking into account volume loss to filtration, improving on previous models which only took into account blood apparent viscosity in calculating pressures throughout the network. Filtration is found to be heterogeneously distributed throughout the glomerular capillary network and is determined by concentration of plasma proteins and surface area of the filtering capillary segments. Hoop stress is primarily concentrated near the afferent arteriole, whereas shear stress is concentrated near the efferent arteriole. Using parameters from glomerular micropuncture studies, conditions of diabetes mellitus (DM), 5/6‐Nephrectomy (5/6‐Nx), and Angiotensin II‐induced hypertension (HTN) are simulated and compared to their own internal controls to assess the changes in mechanical stresses. Hoop stress is increased in all three conditions, while shear stress is increased in 5/6‐Nx, decreased in HTN, and maintained at control levels in DM by the hypertrophic response of the glomerular capillaries. The results indicate that these alterations in mechanical stresses and the consequent release of cytokines by or injury of the glomerular cells may play a significant role in the progression of glomerulopathy in these disease conditions.

## Graphical abstract

We use a novel mathematical model of blood flow and filtration in an anatomically accurate glomerular microvascular network to quantify mechanical stresses and filtration dynamics for each glomerular capillary. Using hemodynamic data from previous literature, we simulate hypertensive, diabetic and renoprival conditions to demonstrate that mechanical stresses are changed due to alterations in pressure and flow in these disease states. Our results indicate that mechanical stress may play a crucial role in the cause and/or progression of glomerular injury in these disease conditions.

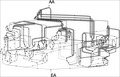

## 1 INTRODUCTION

Through renal autoregulatory control of the afferent arteriole, blood flow and glomerular pressure are tightly regulated (Arendshorst & Navar, 2007; Inscho, 2009). However, in diseases such as diabetes mellitus (DM) and some forms of hypertension, or in the case of loss of functional nephrons due to chronic renal diseases, either the vasoconstrictive response of the afferent arteriole proves inadequate in controlling blood flow and glomerular pressure and/or vasodilatory compensatory responses exhaust the reserve capacity of remaining nephrons to maintain adequate renal function (Carmines, 2010; Hostetter, Olson, Rennke, Venkatachalam, & Brenner, 1981; Hostetter, Rennke, & Brenner, 1982; Inscho, 2009). Similarly, inappropriate activation of the renin‐angiotensin system results in increased afferent and efferent resistance, reducing blood flow into the glomerulus while increasing the glomerular pressure (Franco et al., 2011). These hemodynamic changes result in alterations in single nephron blood flow and glomerular pressure which are believed to alter the magnitudes of shear stress on the glomerular endothelial cells and circumferential hoop stress on the glomerular capillary walls. Mechanical stresses play a crucial role in cell physiology, and inappropriate elevation or reduction in these stresses may lead to glomerulopathy by triggering the release of inflammatory cytokines by endothelial cells (Chiu et al., 2004; Nagel, Resnick, Atkinson, Forbes Dewey, & Gimbrone, 1994; Ohno, Cooke, Dzau, & Gibbons, 1995; Sorescu et al., 2004; Sucosky, Balachandran, Elhammali, Jo, & Yoganathan, 2009; Tsuboi, Ando, Korenaga, Takada, & Kamiya, 1995) and by potentiating hypertrophy and detachment of podocytes (Dessapt et al., 2009; Petermann et al., 2005).

While it is an established concept that mechanical stresses are increased in glomerular capillaries in these diseases, the actual in vivo magnitudes of these stresses are not known primarily due to a lack of robust mathematical models of glomerular mechanics. Numerous models of varying complexity have been developed to estimate the glomerular clearance of fluid and solutes (Deen, Robertson, & Brenner, 1972; Papenfuss & Gross, 1978) however these models assumed the glomerulus to be composed of one to five rigid cylinders filtering in parallel and did not consider the tortuous topology of the glomerular capillary network (Kaczmarek, 1999; Nyengaard & Marcussen, 1993; Shea, 1979; Shea & Raskova, 1984; Wagner, Czymmek, & Hossler, 2006). Few models have been devised which calculate the local filtration dynamics taking into account the actual glomerular anatomy (Lambert et al., 1982; Remuzzi et al., 1992), primarily due to the scarcity of anatomical data traditionally obtained using tedious methods of perfusion fixation and ultrathin sectioning to reconstruct the glomerular capillary network (Shea, 1979; Shea & Raskova, 1984).

Modeling studies that took into account the complexity of the glomerular capillary network topology (Lambert et al., 1982; Remuzzi et al., 1992) used glomerular filtration equations previously derived by Deen et al. (1972) to calculate the filtered volume on each capillary segment in an anatomically accurate glomerular capillary network. Mass and volume conservation laws were used to distribute plasma protein concentration, plasma flow, and erythrocytes through the network. The pressure drop along the length of the network was estimated assuming apparent viscosity of blood to be a function of hematocrit, and the pressure drop was assumed to be linear on each capillary segment without taking into account filtration.

Building on this work, we present a new model that uses a rat glomerular microvascular network geometry obtained through perfusion fixation and ultrathin sectioning of a rat glomerulus in a previous study (Shea, 1979). Our model takes into account the non‐Newtonian properties of blood in calculating shear stresses exerted on the capillary walls (Ferrell et al., 2015) and determining the resistance of the glomerular capillaries (Pries et al., 1994). Our model improves on the previous models by taking into account volume loss to filtration in estimating the pressure profile along each capillary segment without requiring the pressure profile to be linear, and this loss of volume similarly affects the pressures at network nodes. Calibrating our model using hemodynamic data from glomerular micropuncture studies (Franco et al., 2011; Kasiske, O’Donnell, Garvis, & Keane, 1988; Zatz et al., 1986), we investigate the mechanical effect of pathophysiological alterations of renal hemodynamics on the glomerular capillaries, which has not been quantified previously; we use the model to simulate the glomerular hemodynamics in a glomerulus in DM, a remaining glomerulus post 5/6‐Nephrectomy (5/6‐Nx), and a glomerulus in Angiotensin II‐induced hypertension (HTN), and we calculate the glomerular capillary wall shear stress and circumferential hoop stress in these cases.

## 2 MATHEMATICAL MODEL

The mathematical model consists of an anatomical model of the glomerular capillary network, including the network topology, lengths, and diameters of each capillary segment in the network; a filtering capillary model that calculates filtered volume, blood flow, pressure, and the concentration of plasma proteins on the length of each capillary segment; and a network model that iteratively updates the node pressures taking into account filtration of plasma volume and apparent viscosity in determining capillary resistances. Mass balance equations are used to ensure conservation of plasma proteins, erythrocyte volume, and plasma volume on the length of the network. Boundary conditions are enforced by fixing inlet and outlet pressures, setting them equal to mean arterial pressure (MAP) and peritubular capillary pressure, respectively. Afferent and efferent arterioles are represented by fixed resistors at the inlet and outlet of the glomerular network, respectively. The model is steady‐state for the purpose of investigating chronic effects of changes in renal hemodynamics, thus dynamic shifts in renal autoregulation are not considered. The key equations are described below, with details and model derivation included in the appendix.

### 2.1 Glomerular network anatomy

The model anatomical dimensions are derived from a study utilizing perfusion fixation to calculate the topology of, and diameter and length of each capillary within a Sprague Dawley rat glomerulus (Shea, 1979). Based on this data, the rat glomerulus is idealized as a network of straight, cylindrical tubes with prescribed length and diameter, representing filtering capillaries. The network contains 320 segments with a mean diameter of 8.3 μm and mean length of 20.9 μm. Maximum capillary length and diameter are 109 and 21.3 μm, respectively, and minimum capillary length and diameter are 2.5 and 2 μm, respectively. Capillaries branch and coalesce at 193 network nodes. Total glomerular surface area is 1.76 × 10^5^ μm^2^. Originally reported with two efferent arterioles, these vessels are joined at a newly created node. Afferent and efferent arterioles are represented by resistors leading into and out of the network, respectively.

### 2.2 Filtering capillary model

For each capillary segment in the network we solve equations for the pressure profile *p*(*x*), the blood flow *Q*(*x*), the plasma protein concentration *C*(*x*), and the erythrocyte flow *E*, assumed constant on the length of the vessel (Papenfuss & Gross, 1978). We idealize a filtering capillary as a cylindrical tube extending from node *i* to node *j*, with length and diameter *L_ij_* and *D_ij_*, respectively. The resistance to the flow through the capillary is denoted *R_ij_* and we introduce the parameter $R_{\mathrm{ij}}^{f}$ which represents the resistance of the capillary wall to filtration. For *p_ij_*(*x*) the pressure profile on the length of the capillary segment and *p_BS_* Bowman's Space pressure, we define $R_{\mathrm{ij}}^{f}$ such that we may obtain the capillary segment glomerular filtration rate (CSGFR) as a function of the pressure profile:

(1) $${\mathrm{CSGFR}}_{\mathrm{ij}}=\frac{\int_{0}^{L_{\mathrm{ij}}}\left(p_{\mathrm{ij}}\left(x\right)‐p_{\mathrm{BS}}\right)dx}{R_{\mathrm{ij}}^{f}L_{\mathrm{ij}}}.$$

The filtration resistance $R_{\mathrm{ij}}^{f}$ is assumed constant on the length of the capillary and is not fixed but instead will be iteratively updated based on the plasma colloid osmotic and hydrostatic pressure profiles, as described below. For *p_i_* and *p_j_* the node pressures at each end of the capillary, we derive the second order differential equation for the pressure profile *p_ij_*(*x*) along the capillary segment taking into account volume loss via filtration:

(2) $$\frac{d^{2}p_{\mathrm{ij}}}{dx^{2}}\left(x\right)‐a_{\mathrm{ij}}^{2}p_{\mathrm{ij}}\left(x\right)=‐a_{\mathrm{ij}}^{2}p_{\mathrm{BS}}$$

with boundary conditions *p_ij_*(0) = *p_i_* and *p_ij_*(*L_ij_*) = *p_j_*, where $a_{\mathrm{ij}}^{2}=R_{\mathrm{ij}}/R_{\mathrm{ij}}^{f}L_{\mathrm{ij}}^{2}$. We let the capillary segment blood flow

(3) $$Q_{\mathrm{ij}}\left(x\right)=‐\frac{L_{\mathrm{ij}}}{R_{\mathrm{ij}}}\frac{dp_{\mathrm{ij}}}{\mathrm{dx}}\left(x\right).$$

Taking the derivative of Equation 3 and substituting Equation 2 for the second derivative of *p_ij_*(*x*),

(4) $$\frac{dQ_{\mathrm{ij}}}{\mathrm{dx}}\left(x\right)=‐\frac{p_{\mathrm{ij}}\left(x\right)‐p_{\mathrm{BS}}}{R_{\mathrm{ij}}^{f}L_{\mathrm{ij}}}.$$

Following traditional equations of glomerular filtration, we may also represent the change of blood flow on the length of the capillary segment as

(5) $$\frac{dQ_{\mathrm{ij}}}{\mathrm{dx}}\left(x\right)=‐k\pi D_{\mathrm{ij}}\left(p_{\mathrm{ij}}\left(x\right)‐p_{\mathrm{BS}}‐\Pi_{\mathrm{ij}}\left(x\right)\right),$$

for *k* the hydraulic conductivity of the capillary wall, defined as the permeability of the glomerular filtration barrier to water, and Π*_ij_*(*x*) the colloid osmotic pressure as a function of plasma protein concentration *C_ij_*(*x*):

(6) $$\Pi_{\mathrm{ij}}\left(x\right)=2.1C_{\mathrm{ij}}\left(x\right)+0.16C_{\mathrm{ij}}^{2}\left(x\right)+0.009C_{\mathrm{ij}}^{3}\left(x\right)$$

(Papenfuss & Gross, 1978). Assuming mass balance,

(7) $$C_{\mathrm{ij}}\left(x\right)=C_{\mathrm{ij}}\left(0\right)\frac{Q_{\mathrm{ij}}\left(0\right)‐E_{\mathrm{ij}}}{Q_{\mathrm{ij}}\left(x\right)‐E_{\mathrm{ij}}},$$

for *E_ij_* the erythrocyte flow in the capillary segment, assumed constant in *x*. To enforce consistency between Equations 4 and 5, we combine them and integrate on the length of the vessel, so that

(8) $$R_{\mathrm{ij}}^{f}=\frac{\int_{0}^{L_{\mathrm{ij}}}\left(p_{\mathrm{ij}}\left(x\right)‐p_{\mathrm{BS}}\right)dx}{k\pi D_{\mathrm{ij}}L_{\mathrm{ij}}\int_{0}^{L_{\mathrm{ij}}}\left(p_{\mathrm{ij}}\left(x\right)‐p_{\mathrm{BS}}‐\Pi_{\mathrm{ij}}\left(x\right)\right)dx}.$$

With this formulation, we solve for the profiles of the pressure *p_ij_*(*x*), blood flow *Q_ij_*(*x*), plasma protein concentration *C_ij_*(*x*), and colloid osmotic pressure Π*_ij_*(*x*) along the length of each capillary given a constant erythrocyte flow *E_ij_* and hydraulic conductivity *k*. Equation 8 is then used to iteratively update $R_{\mathrm{ij}}^{f}$ as a function of the capillary pressure profile and plasma protein concentration from the previous iteration, as described below.

### 2.3 Filtering network model

In order to assess the boundary conditions of Equations (1), (2), (3), (4), (5), (6), (7), (8), we extrapolate the capillary model to a network of filtering capillaries using mass balance equations. We calculate pressures at each node using a linear system with boundary conditions *p_a_* and *p_e_* at the beginning of the afferent arteriole and the end of the efferent arteriole, respectively. The pressures *p_a_* and *p_e_* are fixed and set equal to MAP and peritubular capillary pressure, respectively (Sgouralis & Layton, 2014), and afferent and efferent resistances are fixed. Due to the relationship between the pressure profile *p_ij_*(*x*) and the resistance to filtration $R_{\mathrm{ij}}^{f}$, filtered volume is taken into account in calculating pressures at each node of the network; once pressures *p_i_* and *p_j_* are calculated, Equations 3 and 4 are used to calculate the blood flows at the boundaries and CSGFR, respectively.

Erythrocyte flow in each capillary segment, *E_ij_* is calculated using the sigmoidal function for distribution of erythrocytes at network nodes in the mesenteric microcirculation derived previously (Pries, Secomb, Gaehtgens, & Gross, 1990). These calculations are dependent upon the hematocrit in the feeding vessel. Hematocrit in a vessel connecting node i to node j is defined as

(9) $${{(H}_{t})}_{\mathrm{ij}}=\frac{E_{\mathrm{ij}}}{\frac{1}{L_{\mathrm{ij}}}\int_{0}^{L_{\mathrm{ij}}}Q_{\mathrm{ij}}\left(x\right)dx}.$$

Afferent erythrocyte flow, *E_A_*, is calculated based on the assumption that 20% of the systemic hematocrit appears in the microvasculature (Lipowsky, Usami, & Chien, 1980). Plasma protein concentration boundary conditions are determined using mass balance equations, assuming mixing of plasma proteins at each network node, with the afferent plasma protein concentration *C_A_* assumed to be known a priori. If we let *K* be the set of nodes *k* upstream of and connected to node *i*, and *J* be the set of nodes *j* downstream of and connected to node *i*, then for all nodes *j* in *J* we define

(10) $$C_{\mathrm{ij}}\left(0\right)=\frac{\sum_{k\epsilon K}C_{\mathrm{ik}}\left(L_{\mathrm{ik}}\right)\left(Q_{\mathrm{ik}}\left(L_{\mathrm{ik}}\right)‐E_{\mathrm{ik}}\right)}{\sum_{k\epsilon K}\left(Q_{\mathrm{ik}}\left(L_{\mathrm{ik}}\right)‐E_{\mathrm{ik}}\right)}.$$

Equation 7 is then used to solve for *C_ij_*(*L_ij_*). With these equations, we calculate the pressure *p_i_* at each node, erythrocyte flow through each capillary segment *E_ij_*, and boundary conditions for the plasma protein concentration *C_ij_*(*x*) for each capillary segment.

### 2.4 Capillary resistance and mechanical stress calculations

To calculate shear stress on the vessel walls, we assume Poiseuille flow through the capillary, taking into account filtration by averaging the flow on the length of the vessel. Assuming Poiseuille flow, the resistance of the capillary

(11) $$R_{\mathrm{ij}}=\frac{128\mu_{\mathrm{ij}}L_{\mathrm{ij}}}{\pi D_{\mathrm{ij}}^{4}}$$

where $\mu_{\mathrm{ij}}$ is the apparent viscosity of the blood as a function of hematocrit:

(12) $$\mu_{\mathrm{ij}}=\mu_{\mathrm{ij}}^{\mathrm{pl}}\lambda \left(D_{\mathrm{ij}},{{(H}_{t})}_{\mathrm{ij}}\right)$$

for $\mu_{\mathrm{ij}}^{\mathrm{pl}}$ the plasma viscosity, taken to be a linear function of plasma protein concentration (Remuzzi et al., 1992), and *λ* a scaling factor that takes into account hematocrit in calculating the apparent blood viscosity (Pries et al., 1994). Similar to the filtration resistance $R_{\mathrm{ij}}^{f}$, the apparent viscosities *μ_ij_* will be iteratively updated based on the plasma protein concentration and erythrocyte flow. Shear stresses on each capillary segment, denoted by *τ* are calculated taking into account loss of flow due to filtration and apparent viscosity of the blood as it passes through the capillary:

(13) $$\tau_{\mathrm{ij}}=\frac{32\mu_{\mathrm{ij}}\frac{1}{L_{\mathrm{ij}}}\int_{0}^{L_{\mathrm{ij}}}Q_{\mathrm{ij}}\left(x\right)dx}{\pi D_{\mathrm{ij}}^{3}}.$$

Hoop stresses on each capillary segment, denoted by *σ* are calculated using the Young‐Laplace equation:

(14) $$\sigma_{\mathrm{ij}}=\frac{D_{\mathrm{ij}}\frac{1}{L_{\mathrm{ij}}}\left(\int_{0}^{L_{\mathrm{ij}}}{(p}_{\mathrm{ij}}\left(x\right)‐p_{\mathrm{BS}})dx\right)}{2t_{\mathrm{ij}}},$$

for glomerular capillary wall thickness *t* assuming an endothelial layer thickness *t* ^e^ of 40 nm (Yamada, 1955), a basement membrane thickness *t* ^bm^ of 149.82 nm in the base case (Dubrulle, Terzi, Gubler, Kleinknecht, & Schaeverbeke, 1992), and an averaged podocyte layer thickness $t_{\mathrm{ij}}^{\text{pod}}$ assuming podocyte foot processes to have a height *h* ^pod^ of 0.3 μm and width *w* ^pod^ of 0.17 μm (Kriz et al., 1994). In general, the thickness of each glomerular capillary wall is estimated assuming a minimum thickness of the podocyte layer $t_{\min}^{\text{pod}}$ due to the presence of the slit diaphragm. Based on these assumptions, we have developed a model of estimating the average glomerular capillary wall thickness:

(15) $$t_{\mathrm{ij}}={t^{e}+t^{\mathrm{bm}}+t}_{\min}^{\mathrm{pod}}+\frac{h^{\mathrm{pod}}}{2}+\frac{1}{\pi D_{\mathrm{ij}}}\int_{0}^{\pi D_{\mathrm{ij}}}\frac{h^{\mathrm{pod}}}{2}\cos\left(\frac{2\pi }{w^{\mathrm{pod}}}\left(\pi D_{\mathrm{ij}}‐\frac{w^{\mathrm{pod}}}{2}\right)\right)dx.$$

Parameters used in model simulations are detailed in Table 1.

**TABLE 1:** Model simulation parameters, including afferent and efferent arteriole resistances, afferent plasma protein *C*_A_, and thickness of the glomerular basement membrane (GBM), Bowman’s Space pressure *p_BS_*, and MAP.

Condition	AA resistance (10^10^ dyn s/cm^5^)	EA resistance (10^10^ dyn s/cm^5^)	C_A_ (g/dl)	GBM thickness (nm)	p_BS_ (mmHg)	MAP (mmHg)	Ref
Base case	5.5	1.0	5.94	149.82	13	115	Dubrulle et al. (1992) and Navar et al. (1986)
Control	4.7	1.3	5.6	138	13	119	Østerby and Gundersen (1980) and Zatz et al. (1986)
DM	2.2	1.0	5.9	162	11	117	
Control	7.2	1.8	5.2	149.82	13	124	Dubrulle et al. (1992) and Kasiske et al. (1988)
5/6‐Nx	2.9	0.8	5.3	146.99	12	138	
Control	5.9	1.5	5.12	149.82	11	115	Dubrulle et al. (1992) and Franco et al. (2011)
HTN	16.1	2.7	5.49	149.82	13	165	

### 2.5 Model algorithm

**FIGURE 1:**
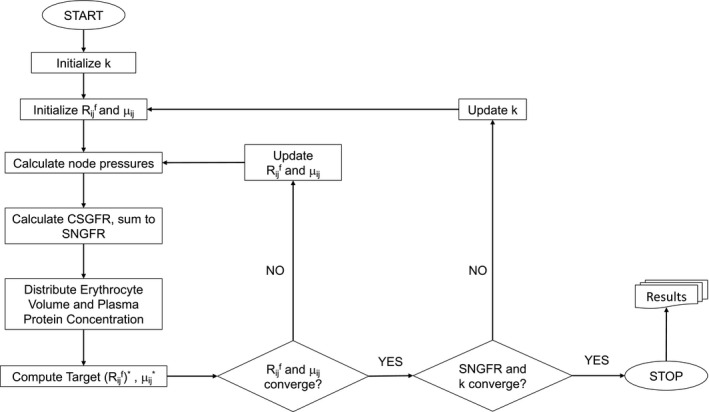
A flowchart of the numerical scheme used to iteratively update filtration resistances $R_{\mathrm{ij}}^{f}$, apparent viscosities *μ_ij_* and hydraulic conductivity *k* until the model converges to a target single nephron GFR (SNGFR) value.

The model equations above are iteratively applied to calculate pressures at nodes, updating vessel resistances, and filtered volume. Additionally, we use an iterative method to determine the hydraulic conductivity *k* for which the model will converge to an expected value of SNGFR given experimental data. We briefly describe the algorithm, with additional details included in the appendix. Equation 8 is used to guess the initial values of $R_{\mathrm{ij}}^{f}$, and apparent viscosities *μ_ij_* are initially set equal to a plasma viscosity of 1.24 cP. Pressures are calculated at each node with inlet and outlet pressure boundary conditions, and Equation 2 is used to calculate the pressure profile on the length of each capillary segment. Filtered volume on the length of each capillary segment is calculated with Equation 1. Using Equations 7 and 10, plasma protein concentration profiles are derived for each capillary segment, and erythrocyte flow is calculated for each capillary segment assuming mass balance. Using Equation 8, $R_{\mathrm{ij}}^{f}$ are updated for each capillary segment. Equation 12 is used to update the apparent viscosity of blood in each segment as a function of vessel hematocrit and diameter, and subsequently update the capillary resistances. In the following iterations, the updated capillary apparent viscosities and filtration resistances are used to recalculate pressures at the network nodes, and the algorithm is repeated until all *μ_ij_* and $R_{\mathrm{ij}}^{f}$ converge to within 0.001% of their values in the previous iteration. If both *μ_ij_* and $R_{\mathrm{ij}}^{f}$ converge, we check to see if the model SNGFR converges to the SNGFR reported in the experimental study to which we are fitting the model and if k converges. We update *k* if convergence is not reached. A schematic of the algorithm is provided in Figure 1.

## 3 RESULTS

We assume base case conditions of a mean glomerular capillary pressure (*P*_GC_) of 50 mmHg and SNGFR of 30 nl/min (Navar, Bell, & Evan, 1986). We calculate network *P*_GC_ by taking the mean of the averaged pressures of each glomerular capillary. We use a *Q*_A_ of 129 nl/min (Franco et al., 2011; Kasiske et al., 1988; Zatz et al., 1986) and an afferent plasma protein concentration (*C*_A_) of 5.94 g/dl to enforce an average colloid osmotic pressure Π of 25 mmHg (Navar et al., 1986). We calculate the average colloid osmotic pressure as in glomerular micropuncture experiments, by taking the average of the inlet and outlet colloid osmotic pressures. Using the base case conditions, we conduct a sensitivity analysis of salient parameters and examine the spatial distribution of mechanical stresses and filtration throughout the glomerular capillary network. Afferent and efferent resistances are adjusted until the mean of the average pressure on each capillary segment equals the target *P*_GC_ and plasma flow through the afferent arteriole equals the target *Q*_A_. Hydraulic conductivity is adjusted until the model SNGFR equals the target SNGFR. In addition to the base case, we employ this strategy to simulate disease conditions.

### 3.1 Sensitivity analysis of glomerular mechanical stresses

**FIGURE 2:**
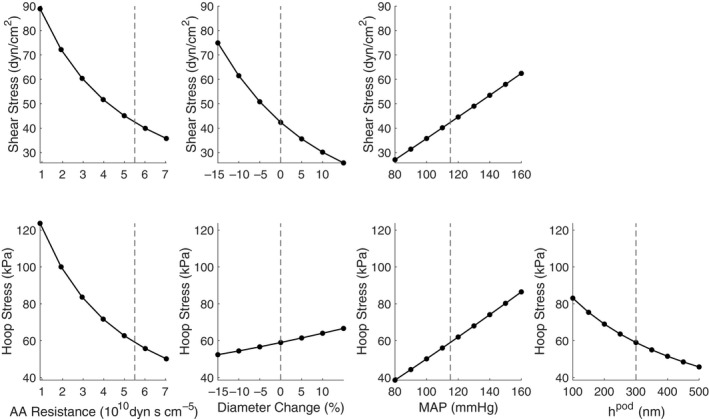
Analysis of the sensitivity of mechanical stresses to variance of afferent arteriole (AA) resistance, percent alteration in all glomerular capillary diameters, mean arterial pressure MAP, and podocyte foot process height *h* ^pod^. Vertical dashed lines indicate the values used in the base case.

We conduct a sensitivity analysis to evaluate the effect of varying afferent arteriole resistance, glomerular capillary diameters, and MAP on mechanical stresses exerted on the glomerular capillaries (Figure 2). Increases in afferent arteriole resistance reduce glomerular pressure and flow, thus reducing both shear and hoop stresses. Small changes in the glomerular capillary diameters directly influence the mean network shear stress, *τ* and hoop stress, *σ*, according to Equations 13 and 14. As would be expected, increasing the inlet pressure increases *Q*_A_ and *P*_GC_, with consequent increases in shear and hoop stresses. To facilitate the comparison to experimental studies, the afferent arteriole resistance is calculated as in micropuncture experiments assuming no reduction of hematocrit in the microvasculature (Lipowsky et al., 1980). Alteration of the podocyte foot process height results in significant changes in hoop stress throughout the network, highlighting the importance of podocytes in counteracting capillary wall distention (Kriz et al., 1994).

**FIGURE 3:**
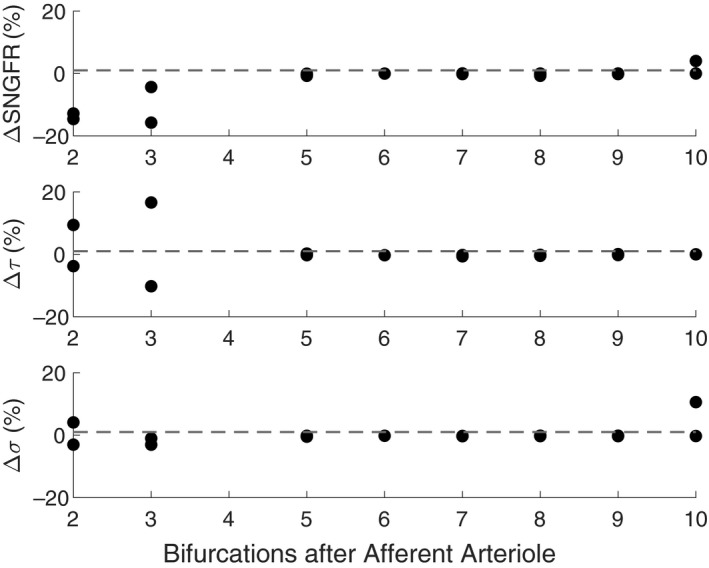
Analysis of the sensitivity of SNGFR, mean network shear stress *τ*, and mean network hoop stress *σ* to reduction of a single capillary segment diameter to 0.1 μm, based on location of the segment in the network. Changes in SNGFR, *τ*, and *σ* are represented by % change over baseline.

Minor alterations in network topology are considered by setting the diameter of individual capillaries to 0.1 μm to evaluate the effect of this change on SNGFR and magnitudes of mechanical stresses exerted throughout the network (Figure 3). Distance of a particular capillary segment from the afferent arteriole is ascertained by computing the number of bifurcations on the shortest path between the afferent arteriole and the source node of the capillary segment (*x*‐axis in Figure 3). Based on where the altered segments lie in relation to the afferent arteriole, it is apparent that altering the diameter of the segments closest to the afferent and efferent arterioles has the greatest effect on filtration and mechanical stresses in the glomerular capillaries.

### 3.2 Spatial distribution of mechanical stresses and filtration

**FIGURE 4:**
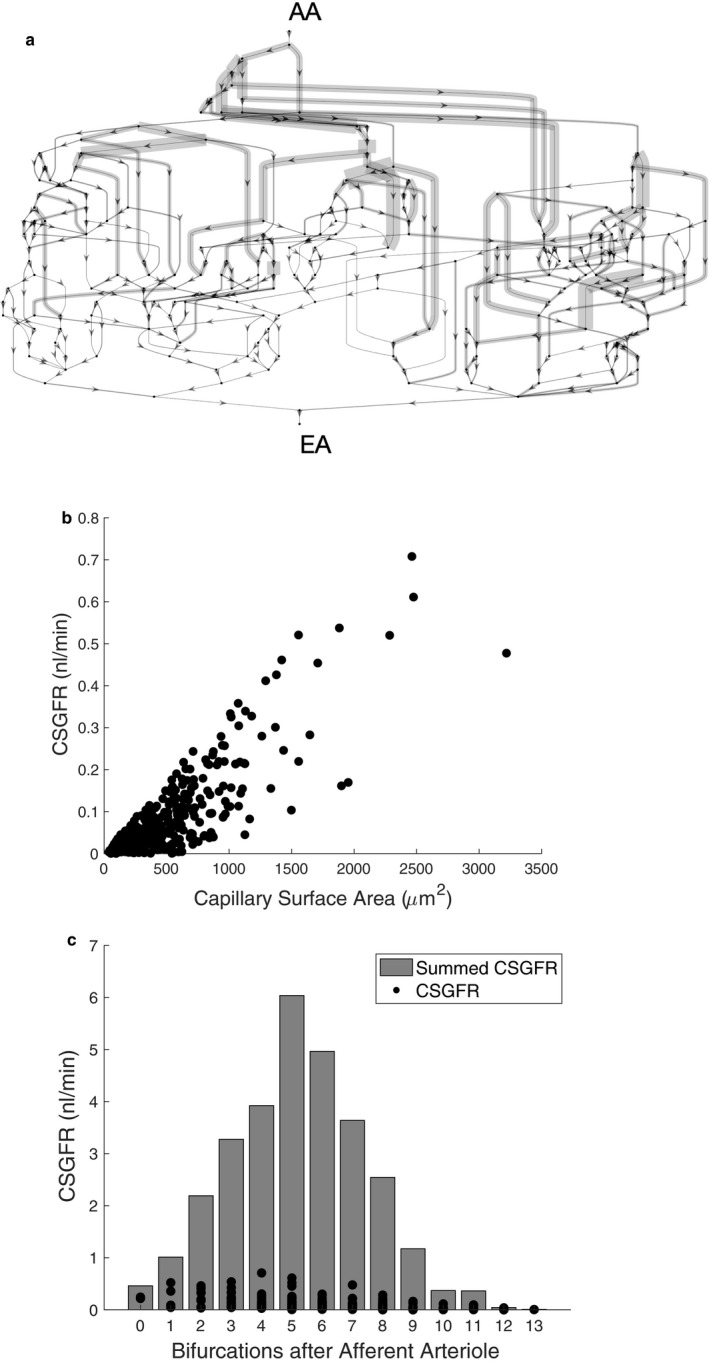
(a) Spatial distribution of capillary segment glomerular filtration rate (CSGFR) throughout the glomerular capillary network. CSGFR values are graphed on the glomerular network topology to facilitate comparison between CSGFRs at different locations in the network. Thickness of each segment is proportional to the filtration rate. AA––afferent arteriole, EA––efferent arteriole. (b) CSGFR is plotted against capillary surface area to determine the cause of heterogenous filtration throughout the capillary network. (c) Individual and summative CSGFRs are graphed as a function of distance of the capillary segment from the afferent arteriole

**FIGURE 5:**
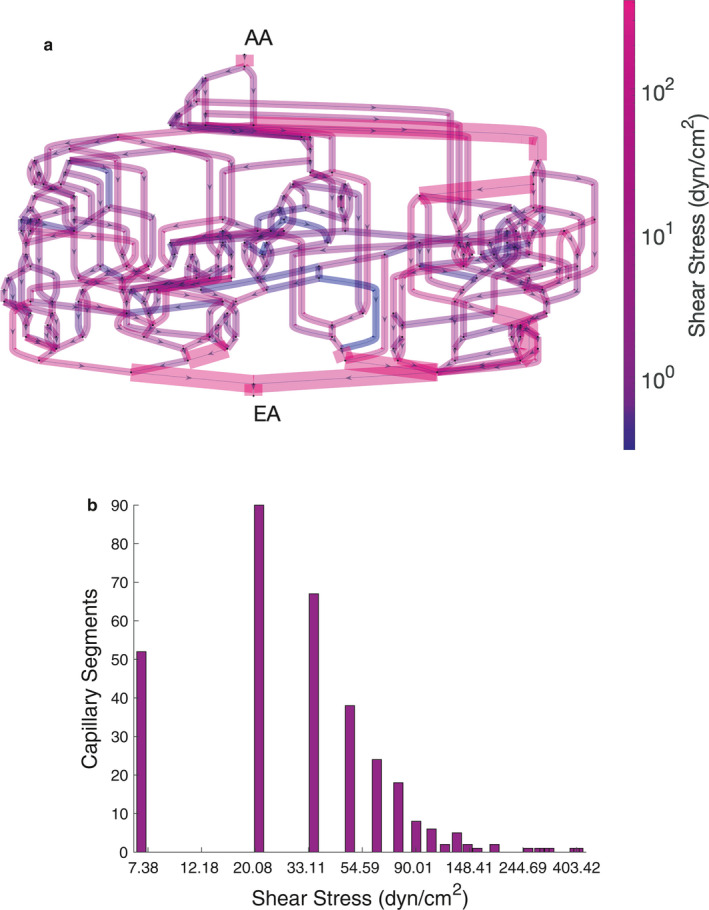
(a) Shear stress magnitudes and locations within the glomerular capillary network. A scale bar is used to indicate shear stress magnitude on a log scale, and segments with shear stress over 100 dynes/cm^2^ are of greater width. AA––afferent arteriole, EA––efferent arteriole. (b) A histogram details frequency of shear stress values throughout the network.

**FIGURE 6:**
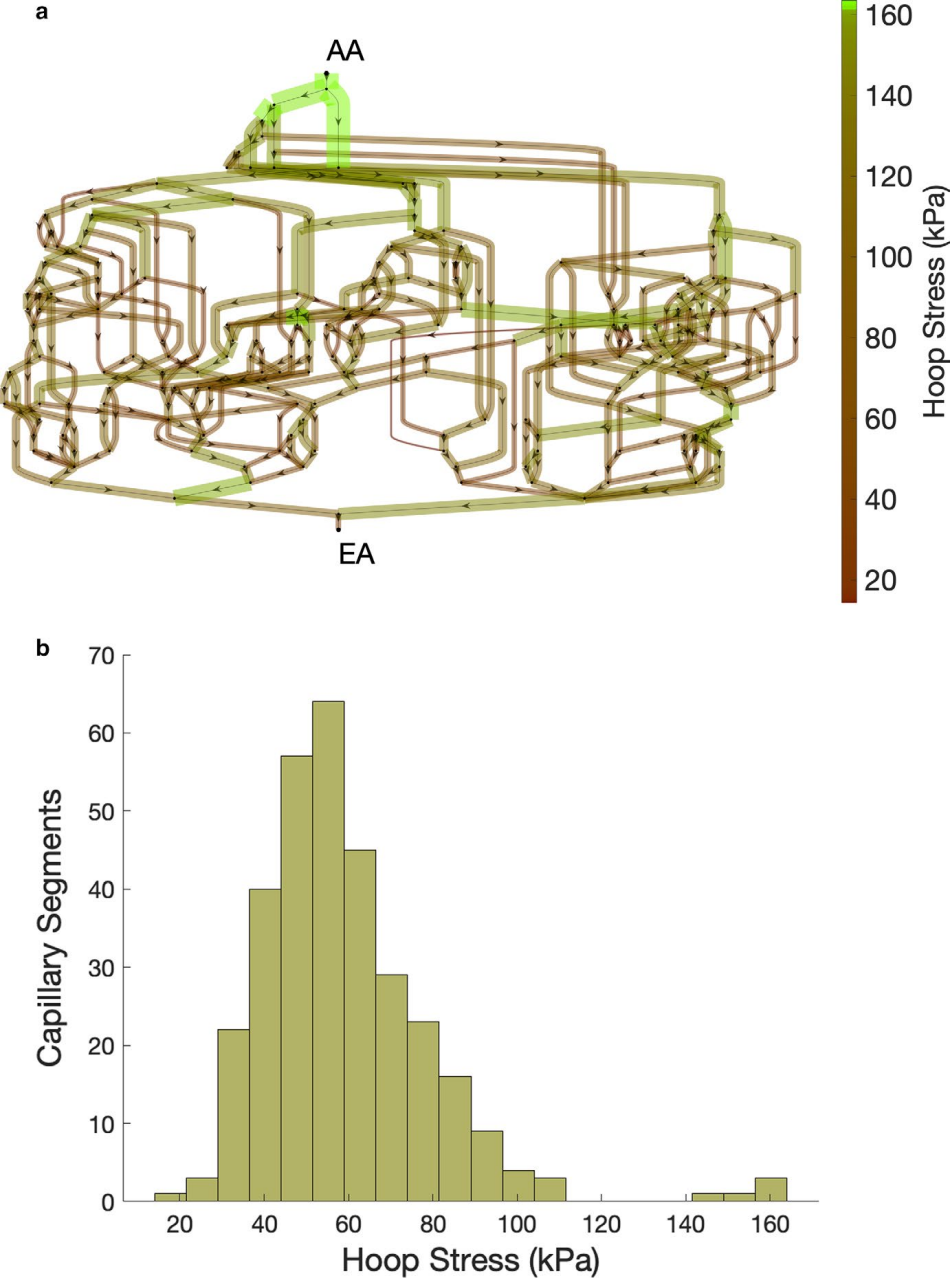
(a) Circumferential hoop stress magnitudes and location within the glomerular capillary network, where the scale bar at right indicates magnitude of hoop stress. AA––afferent arteriole, EA––efferent arteriole. (b) A histogram to represent the frequency of hoop stress magnitudes throughout the network.

Using base case conditions, we examine the spatial distribution of filtration (Figure 4) and mechanical stresses (Figures 5 and 6) throughout the glomerular capillary network. We demonstrate that filtration is heterogeneously distributed throughout the glomerular capillary network (Figure 4a) and is heavily influenced by individual glomerular capillary surface areas (Figure 4b). While most of the capillaries closest to the afferent arteriole filter due to the lower concentration of plasma proteins, the summative surface area is highest in the “middle” of the glomerulus where the larger number of capillary segments increases the summative filtration in this region (Figure 4c). Capillaries furthest downstream from the afferent arteriole are rendered nonfiltering as plasma proteins concentrate to the point where plasma colloid osmotic pressure equalizes the hydrostatic pressure gradient, causing filtration to cease.

Shear stresses are generally concentrated closer to the efferent arteriole than the afferent arteriole due to the concentration of plasma proteins and hematocrit which increase the plasma viscosity closer to the efferent arteriole (Figure 5a). Shear stress also appears to be concentrated within individual lobules or groupings of glomerular capillaries, with low magnitudes in the cross‐cutting anastomoses between the lobules. Mean network shear stress is 42.4 dyn/cm^2^, with a minimum of 0.3 dyn/cm^2^ and a maximum of 399.7 dyn/cm^2^, demonstrating the large variance of shear stress throughout the glomerular capillary network. In contrast to the concentration of shear stresses near the efferent arteriole, hoop stresses are concentrated near the afferent arteriole due to the larger diameters of the vessels that branch off of the afferent arteriole upon entering the glomerulus (Figure 6a). The mean hoop stress on the network is 59.0 kPa, with a minimum of 14.3 kPa and a maximum of 160.7 kPa. Given that 1 kPa is equal to 7.5 mmHg, the large hoop stress relative to the pressure drop across the capillary walls is caused by the thinness of the glomerular filtration barrier in comparison to the capillary diameter, where the wall thickness is in the order of nm while the diameter is in the order of μm.

### 3.3 Hydraulic conductivity and filtration coefficient *K_f_*

**FIGURE 7:**
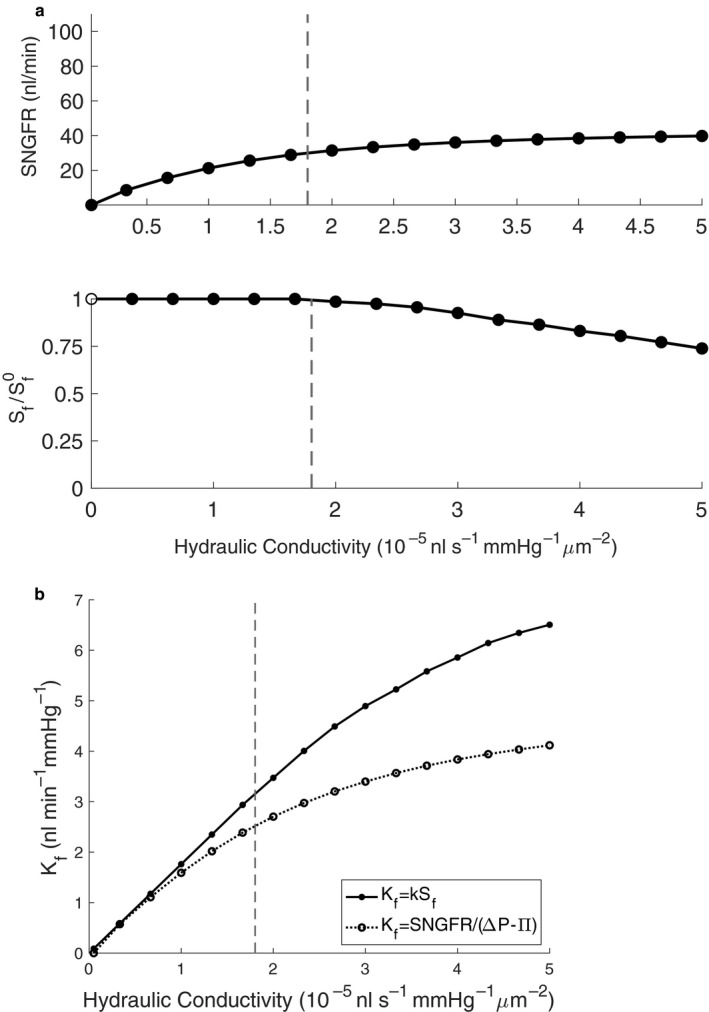
(a) An analysis of the sensitivity of SNGFR and filtering surface area S_f_ to changes in hydraulic conductivity, for *S_f_* normalized to the total surface area $S_{f}^{0}$ equal to 1.76 × 10^5^ μm. (b) Comparison of filtration coefficient *K_f_* calculations, including taking the product of the hydraulic conductivity and the filtering surface area, and the indirect use of the glomerular filtration equation to estimate *K_f_*. The vertical dashed line indicates the value of hydraulic conductivity used in the base case

In conducting a sensitivity analysis of SNGFR and filtering surface area (*S_f_*) to hydraulic conductivity (Figure 7a), we see that hydraulic conductivity *k* has a negative relationship with the filtering surface area, due to the upstream concentration of plasma proteins as shown in Figure 4. The filtration coefficient *K_f_* is equal to the product of the hydraulic conductivity and the filtering surface area of the glomerulus. However, *K_f_* is typically calculated by dividing SNGFR by the average effective filtration pressure, according to the glomerular filtration equation:

(16) $$\mathrm{SNGFR}=K_{f}\left(\Delta P‐\Pi \right).$$

We compare both methods for calculating *K_f_* (Figure 7b), and demonstrate that both methods approach a plateau of *K_f_* at large hydraulic conductivities, corresponding to filtration equilibrium. Using the glomerular filtration equation underestimates *K_f_* in comparison to the direct multiplication of the hydraulic conductivity with the filtering surface area, which can be attributed to an underestimation of the average colloid osmotic pressure on the network by averaging afferent and efferent colloid osmotic pressures to calculate Π, when the colloid osmotic pressures in individual capillaries may exceed this value due to heterogenous filtration throughout the network.

### 3.4 Disease condition simulations

We simulate three disease conditions that alter glomerular hemodynamics: DM, 5/6‐Nx, and HTN. By optimizing the model afferent and efferent resistances, we match the model afferent plasma flow *Q*_A_ and mean glomerular capillary pressure *P*_GC_ to hemodynamic data obtained from glomerular micropuncture studies for both control and disease conditions, assuming inlet pressure to be equal to MAP (Franco et al., 2011; Kasiske et al., 1988; Zatz et al., 1986). We then optimize the hydraulic conductivity to match the model SNGFR to that of the respective studies.

In DM, hyperglycemia acts directly on afferent arteriole vascular smooth muscle cells to reduce afferent and efferent resistances thereby increasing the glomerular pressure and blood flow (Carmines, 2010; Zatz et al., 1986). In the case of 5/6‐Nx, significant loss of functional nephrons results in diversion of blood to the remaining kidney mass, substantially increasing blood flow and pressure in the remnant glomeruli (Kasiske et al., 1988). In the HTN case, angiotensin II constricts both afferent and efferent arterioles, resulting in decreased afferent blood flow and a slightly increased glomerular pressure (Franco et al., 2011). In addition to hemodynamic alterations, the glomerular capillaries undergo structural changes. In DM, capillaries dilate, increasing their diameters by 14% (Østerby & Gundersen, 1980). Increased production of extracellular matrix components by mesangial cells and podocytes results in a thickened basement membrane, from 138 nm in control conditions to 162 nm in DM (Østerby & Gundersen, 1980). In the case of 5/6‐Nx, capillary diameters are increased by 23% (Bidani, Mitchell, Schwartz, Navar, & Lewis, 1990), while the basement membrane does not thicken (Dubrulle et al., 1992). No structural changes are assumed to occur in HTN.

Common across all three disease states is the increase in glomerular pressure over their respective controls, which consequently raises the mean hoop stress on the glomerular capillaries (Table 2). The increase in afferent plasma flow in DM and 5/6‐Nx explains the increased mean network shear stress in these disease conditions over their respective controls, however the shear stress in DM is increased only slightly due to the remodeling of the diabetic rat glomerulus (Østerby & Gundersen, 1980) which increases the glomerular capillary diameters. This remodeling is even more pronounced in the remnant glomeruli post 5/6‐Nx (Bidani et al., 1990), however the massive increase in blood flow to the remnant glomeruli still results in a significantly increased shear stress. In the case of HTN, a reduction in afferent blood flow reduces mean shear stress from the control condition.

**TABLE 2:** Disease condition simulations of DM, 5/6‐Nx, and HTN, with their respective controls. Hemodynamic data, including glomerular capillary pressure *P*_GC_, afferent plasma flow *Q*_A_, and SNGFR, is obtained from the listed references and reproduced by the model via parameter optimization.

Condition	Mean *P*_GC_ (mmHg)	*Q*_A_ (nl/min)	SNGFR (nl/min)	Hydraulic conductivity (10^–5^ nl/min mmHg^−1^ μm^−2^)	Filtering surface area (μm^2^)	Mean shear stress (dyn/cm^2^)	Mean hoop stress (kPa)	Ref
Control	52	154	45.9	2.3	176,213	49	66	Zatz et al. (1986)
DM	63	268	81.6	1.9	200,882	54	93	
Control	51	112	35.5	1.6	176,213	34	62	Kasiske et al. (1988)
5/6‐Nx	57	309	97.0	2.4	217,271	43	91	
Control	49	122	36.2	1.6	176,213	36	61	Franco et al. (2011)
HTN	55	76	23.1	0.85	176,213	24	69	

## 4 DISCUSSION

We developed a novel model of blood flow and filtration in the glomerular microvascular network that takes into account blood rheology, blood phase separation at network nodes, plasma protein concentration, and colloid osmotic pressure. Previous models rely on an assumption that the pressure profile is linear on the length of each capillary, with capillary resistance dependent upon the blood apparent viscosity (Lambert et al., 1982; Remuzzi et al., 1992). Our model improves on these models by taking into account loss of volume due to filtration, thereby allowing for nonlinear pressure profiles. By taking into account the complex anatomical dimensions of the glomerular capillary network, our model allows for in‐depth investigations into the chemical and mechanical microenvironment of glomerular cells and the contribution of different populations of the glomerular capillaries to overall glomerular function. We expand on previous work by applying our model to investigate the mechanical consequences of pathophysiological alterations in renal hemodynamics.

In baseline conditions our model median shear stress is 31.2 dyn/cm^2^, which matches closely with the shear stress of 31.7 dyn/cm^2^ estimated experimentally by Ferrell et al. (2015), providing validation of our mechanical predictions. Our sensitivity analysis indicates that of particular importance are glomerular capillary diameters in estimating mechanical stresses; increasing the glomerular capillary diameters results in a reduced mean shear stress and an increased hoop stress on the glomerular capillary walls. Our sensitivity analysis also suggests that network topology plays an important role in determining the mechanical stresses and filtration in the glomerulus (Figure 3). Altering individual capillary diameters close to the afferent or efferent arterioles results in substantial diversions of blood flow which may alter the mean network shear stress. In this case reduction of the diameters is considered, which leads to a reduction of SNGFR if the altered segment is close to the afferent arteriole. This sensitivity demonstrates that both function and mechanics are highly dependent upon anatomical parameters, particularly the number and diameter of capillaries branching off of the afferent arteriole.

We demonstrate that filtration is heterogeneously distributed throughout the glomerular capillary network, that some segments are sent into a nonfiltering state by nature of the filtration that occurs upstream, and that some segments filter more than others due to their larger surface area. Because there is more cumulative surface area in the “middle” of the glomerulus, the summative filtration is highest after several generations of branching after the afferent arteriole, which has not been demonstrated by previous models that do not take the glomerular anatomy into account and thus assume the highest magnitude of filtration occurs nearest to the afferent arteriole (Deen et al., 1972). Nonfiltering surface area is a result of increasing concentration of plasma proteins upstream. As the network hydraulic conductivity is increased, capillaries further from the afferent arteriole will see an increase in plasma protein concentration and thus colloid osmotic pressure, explaining the relationship between hydraulic conductivity and S_f_ seen in the sensitivity analysis (Figure 7a). The reciprocal relationship of the hydraulic conductivity and the filtering surface area in addition to the plateau of the SNGFR in the sensitivity analysis suggests the capability of the glomerulus to maintain function in the event of physiological or pathological alterations or perturbations of the hydraulic conductivity of the glomerular capillary wall.

The relationship between hydraulic conductivity and filtering surface area is further explored in Figure 7b, in which the filtration coefficient *K_f_* is calculated directly through taking the product of hydraulic conductivity and filtering surface area, and indirectly through the glomerular filtration equation. The former calculation overestimates the latter due to the simplifying assumption in the latter that average colloid osmotic pressure may be estimated by taking the average of the inlet and outlet colloid osmotic pressures; by taking the average of the colloid osmotic pressures in each capillary of the network, the model average colloid osmotic pressure exceeds this estimation by roughly 3 mmHg, which justifies the greater value of *K_f_* calculated by taking the product of the hydraulic conductivity and the filtering surface area.

The model predicts that in DM, shear stress is largely controlled by the remodeling of the glomerular capillaries which increases the capillary diameter, indicating that this remodeling acts as a protective mechanism to reduce shear stress on the endothelium. However, this increase in capillary diameter leaves the capillary more vulnerable to hoop stresses generated by the increased *P*_GC_. While the glomerular basement membrane thickens due to increasing production of ECM components (Østerby & Gundersen, 1980), the overall thickness of the glomerular capillary wall does not increase proportionally to the increase in diameter thus the hoop stress increases by 41%. Diabetic nephropathy is characterized by a loss of podocytes and hypertrophy of the remaining podocytes (Pagtalunan et al., 1997), a finding consistent with the podocyte's response to stretch in vitro (Dessapt et al., 2009; Petermann et al., 2005). While our model does not consider mechanical strain, increased levels of chronic hoop stress may play a role in the reorganization of the podocyte actin cytoskeleton as the podocyte undergoes structural changes to counteract the distention of the glomerular capillary wall (Kriz et al., 1994). The increased capillary diameter also subjects podocytes to increased risk of hypertrophy and detachment due to hoop stresses resulting from transient rises in glomerular pressure.

In our simulations of a glomerulus in the remaining kidney mass post‐5/6‐nephrectomy, both shear stress and hoop stress are increased, the former as a result of a substantial increase in afferent blood flow and the latter a result of a lack of thickening of the glomerular basement membrane, increased intraglomerular pressure, and increased glomerular capillary diameters. Despite this increase in diameter, mean capillary shear stress is increased by 26% in 5/6‐Nx over control. This finding is in opposition to results by Ferrell et al. (2015) who showed that the increase in capillary diameter results in a combined effect of reduction in shear rate and apparent viscosity. This discrepancy can be attributed to the fact that Ferrell et al. imaged the rat glomeruli 2 weeks after renal ablation, whereas the hemodynamic data used in our simulation was gathered at 4 weeks after renal ablation. Ferrell et al. measured a mean increase of 16% in glomerular capillary diameter and a 110% increase in blood flow post‐5/6‐Nx, however in our simulation we assume a 23% increase in glomerular capillary diameter based on a morphometric analysis of remnant glomeruli 4 weeks post 5/6‐Nx (Bidani et al.,^1990^), and a 180% increase in blood flow based on our hemodynamic data (Kasiske et al., 1988). Results from Ferrell et al. indicate that glomerular capillary remodeling may compensate for the increased blood flow for a short period after the renal ablation, but our results show that even with further dilation the capillaries are subjected to higher shear stresses in the long‐term. Based on data of Ferrell et al. (2015) we increased the capillary diameters by 16.1% and the AA blood flow by 110% in our model and found an 18% increase in shear stress (data not shown). This suggests that asymmetric distribution of erythrocytes and plasma protein concentration, both not considered in the work of Ferrell et al., play an important role in determining the degree to which mean network shear stress changes in disease conditions.

In response to laminar shear stress at magnitudes comparable to the mean shear stress calculated in our 5/6‐Nx simulation, endothelial cells increase production of TGF‐β1, and endothelial cell TGF‐β1 mRNA increases proportionally to the increase in shear stress magnitude (Ohno et al., 1995). Cell surface expression of ICAM‐1 follows a similar pattern, whereby ICAM‐1 expression increases with increasing shear stress magnitude (Tsuboi et al., 1995). Given the profibrotic effect of TGF‐β1 and the role of ICAM‐1 in initiating the inflammatory process, the increased shear stress––in combination with increases in hoop stress––may play a significant role in progression of glomerulopathy in the remaining glomeruli in severe renoprival conditions.

In our simulations of HTN, we see that hoop stresses are modestly increased by roughly 13%, whereas shear stresses are decreased by 33% due to the increasing afferent and efferent arteriole resistances which in turn reduce blood flow into the glomerulus. While the increased hoop stress is significant, the reduction in shear stress may also play a role in perpetuating injury of the glomerulus through the action of endothelin; microvascular endothelial cells secrete increased levels of endothelin at shear stresses under a threshold of 15 dynes/cm^2^, and reduce secretion at higher levels (Wang et al., 2002). In control conditions, our model indicates that 23% of the capillaries are subjected to shear stresses below this threshold. With the reduction of flow in the HTN condition, the fraction of capillaries within the range of increased endothelin secretion rises to 43%. Independent of hypertension, endothelin increases glomerular permeability to albumin, and selective antagonists of the ET_A_ receptor attenuate this effect (Saleh, Boesen, Pollock, Savin, & Pollock, 2010), indicating that endothelin, and thus a reduction of shear stress, may play a significant role in the progression of glomerulopathy in hypertension. According to our model results, lower shear stresses primarily occur in anastomoses between the lobules and regions closer to the afferent arteriole, which may in turn result in transport of secreted endothelin to other regions of the glomerulus by the blood flow.

Our sensitivity analysis details the importance of the network topology and vessel diameters in calculating mechanical stresses and glomerular dynamics, and the results of the simulation studies are sensitive to the hemodynamic data gathered from glomerular micropuncture studies. Additional studies must incorporate more anatomical and hemodynamic data to build a better understanding of the variance of mechanical stress and function between different glomeruli.

Additional important assumptions of our model include the assumption that the capillary segments have constant diameter along their length, which has a significant effect on capillary resistance (Iordache & Remuzzi, 1995), and the assumption that shear stress may be quantified assuming Poiseuille flow which does not take into account complexities of fluid flow arising from filtration through the capillary wall. The steady‐state model used in this study does not consider temporal dynamics of renal autoregulation and consequent alterations in SNGFR. These limitations make obvious the need for additional study of glomerular function and mechanics using more sophisticated mathematical models.

We demonstrate that shear stress and hoop stress are concentrated near the efferent arteriole and afferent arteriole, respectively. This novel finding correlates with perihilar glomerular sclerosis, a common form of glomerular sclerosis wherein scarring is primarily visible at the vascular pole, at which both afferent and efferent arterioles are located (Fogo, 2015). The model results, combined with previous literature regarding the effect of mechanical stresses on endothelial cells and podocytes, indicate that changes in mechanical stress may play a role in the cause and/or progression of glomerulosclerosis in conditions in which glomerular hemodynamics are significantly altered, such as DM, HTN, or the loss of renal mass.

## DISCLOSURES

All authors declare no competing interests.

## AUTHOR CONTRIBUTIONS

O.R. and R.C. developed the mathematical model formulation. O.R. conducted model experiments and data analysis. L.G.N. advised on parameter selection and physiological relevance of model results. O.R. wrote manuscript. O.R, R.C, and L.G.N. edited the manuscript.

## Figures and Tables

**FIGURE 8:**
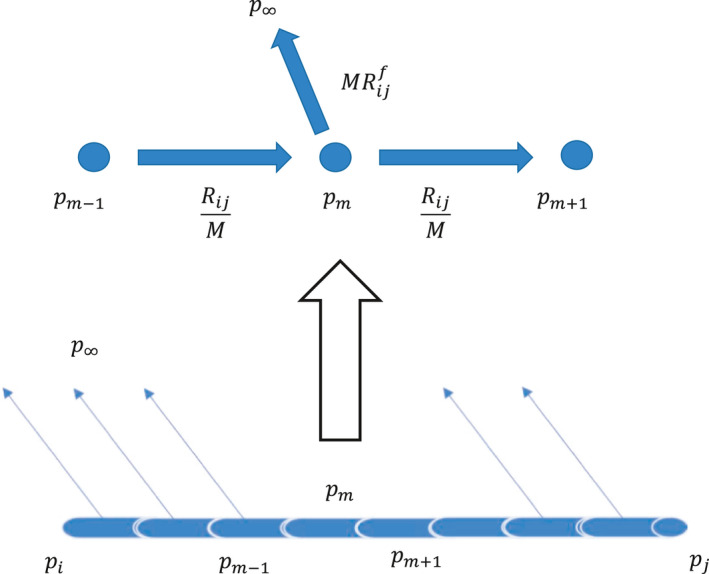
A schematic representation of volumetric flux balance at one point *m* out of *M* total points along the length of the capillary between nodes *i* and *j*. Pressure at point m is denoted by *p_m_*, resistance of the capillary is denoted *R_ij_*, and the resistance to filtration is assumed constant on the length of the vessel and is denoted by $R_{\mathrm{ij}}^{f}$

## APPENDIX 1

### Capillary model derivation

We aim to develop a model that allows for the solution of pressures at network nodes, and for calculation of the pressure profile on the length of each capillary segment. We assume a capillary spans from node *i* to node *j* and is of length *L_ij_*. The capillary is discretized into *M* segments, each with length *L_ij_*/*M*. If the capillary has resistance *R_ij_*, each segment will in turn have resistance *R_ij_*/*M*. The filtration resistance $R_{\mathrm{ij}}^{f}$ is introduced such that each segment filters with filtration resistance *M* $R_{\mathrm{ij}}^{f}$. For *m* = 1, 2, 3, … *M*−1, we denote pressures *p_m_* to be pressures along the length of the capillary, with *p*_0_ = *p_i_* and *p_M_* = *p_j_* (Figure 8). The pressure outside the capillary, denoted *p*_∞_, is assumed constant on the length of the capillary segment. Volume flux balance at m is such that:

(17) $$\frac{p_{m‐1}‐p_{m}}{\frac{R_{\mathrm{ij}}}{M}}+\frac{p_{m+1}‐p_{m}}{\frac{R_{\mathrm{ij}}}{M}}+\frac{p_{\infty }‐p_{m}}{{\mathrm{MR}}_{\mathrm{ij}}^{f}}=0.$$

This implies that

(18) $$‐M^{2}p_{m‐1}+\left(2M^{2}+\frac{R_{\mathrm{ij}}}{R_{\mathrm{ij}}^{f}}\right)p_{m}‐M^{2}p_{m+1}=\frac{R_{\mathrm{ij}}}{R_{\mathrm{ij}}^{f}}p_{\infty }$$

writing in terms of *h* = *L*/*M*, and letting $a_{\mathrm{ij}}^{2}=R_{\mathrm{ij}}/L_{\mathrm{ij}}^{2}$ $R_{\mathrm{ij}}^{f}$,

(19) $$\frac{p_{m‐1}‐2p_{m}+p_{m+1}}{h^{2}}‐a_{\mathrm{ij}}^{2}p_{m}=‐a_{\mathrm{ij}}^{2}p_{\infty }$$

which is a discretization of

(20) $$\frac{d^{2}p_{\mathrm{ij}}}{dx^{2}}\left(x\right)‐a_{\mathrm{ij}}^{2}p_{\mathrm{ij}}\left(x\right)=‐a_{\mathrm{ij}}^{2}p_{\infty }$$

where *p_ij_* is the pressure profile from node *i* to node *j*. We solve equation 20 with boundary conditions *p_ij_*(‐*L_ij_*/2) = *p_i_* and *p_i_*_j_(*L_ij_*/2) = *p_j_*:

(21) $$p_{\mathrm{ij}}\left(x\right)=p_{\infty }+\left(\frac{p_{i}+p_{j}}{2}‐p_{\infty }\right)\frac{\cosh\left(a_{\mathrm{ij}}x\right)}{\cosh\left(\frac{a_{\mathrm{ij}}L_{\mathrm{ij}}}{2}\right)}+\left(\frac{p_{j}‐p_{i}}{2}\right)\frac{\sinh\left(a_{\mathrm{ij}}x\right)}{\sinh\left(\frac{a_{\mathrm{ij}}L_{\mathrm{ij}}}{2}\right)}.$$

with this formulation for *p_ij_*, and using Equation 3 we see that *Q_ij_*(*x*), the blood flow through capillary *ij*, is:

(22) $$Q_{\mathrm{ij}}\left(x\right)=‐\frac{L_{\mathrm{ij}}}{R_{\mathrm{ij}}}\left(\frac{p_{i}+p_{j}}{2}‐p_{\infty }\right)\frac{a_{\mathrm{ij}}\sinh\left(a_{\mathrm{ij}}x\right)}{\cosh\left(\frac{a_{\mathrm{ij}}L_{\mathrm{ij}}}{2}\right)}‐\frac{L_{\mathrm{ij}}}{R_{\mathrm{ij}}}\left(\frac{p_{j}‐p_{i}}{2}\right)\frac{a_{\mathrm{ij}}\cosh\left(a_{\mathrm{ij}}x\right)}{\sinh\left(\frac{a_{\mathrm{ij}}L_{\mathrm{ij}}}{2}\right)}.$$

In deriving the capillary model, we assume that the pressure outside the capillary, *p*_∞_, is independent of *x*. To apply the capillary model to estimate the filtration in a glomerular capillary, we must incorporate the plasma protein concentration and colloid osmotic pressure. To do so, we let *p*_∞_ = *p_BS_* and calculate the resistance to filtration $R_{\mathrm{ij}}^{f}$ as a function of the average colloid osmotic pressure on the length of the capillary segment, implemented in Equation 8. This strategy guarantees that the amount of filtered volume is consistent with the average colloid osmotic pressure on the length of the glomerular capillary and the hydraulic conductivity of the glomerular capillary wall. Because resistance to filtration is a function of the pressure profile, an iterative method is necessary to update $R_{\mathrm{ij}}^{f}$ until every value of $R_{\mathrm{ij}}^{f}$ converges.

### Network model derivation

We wish to conserve volume flow at nodes of the network taking into account loss of blood flow along the length of each capillary segment. Using the equations for flow and pressure derived above, for segment *ij* connecting nodes *i* and *j*, flow at nodes *i* and *j*, denoted *Q_i_* = *Q_ij_*(−*L*/2) and *Q_j_* = *Q_ij_*(*L*/2), respectively, are computed as follows:

(23) $$Q_{i}=\frac{L_{\mathrm{ij}}}{R_{\mathrm{ij}}}\left(\frac{p_{i}+p_{j}}{2}‐p_{\mathrm{BS}}\right)a_{\mathrm{ij}}\tanh\left(\frac{a_{\mathrm{ij}}L_{\mathrm{ij}}}{2}\right)‐\frac{L_{\mathrm{ij}}}{R_{\mathrm{ij}}}\left(\frac{p_{j}‐p_{i}}{2}\right)\frac{a_{\mathrm{ij}}}{\tanh\left(\frac{a_{\mathrm{ij}}L_{\mathrm{ij}}}{2}\right)},$$

(24) $$Q_{j}=‐\frac{L_{\mathrm{ij}}}{R_{\mathrm{ij}}}\left(\frac{p_{i}+p_{j}}{2}‐p_{\mathrm{BS}}\right)a_{\mathrm{ij}}\tanh\left(\frac{a_{\mathrm{ij}}L_{\mathrm{ij}}}{2}\right)‐\frac{L_{\mathrm{ij}}}{R_{\mathrm{ij}}}\left(\frac{p_{j}‐p_{i}}{2}\right)\frac{a_{\mathrm{ij}}}{\tanh\left(\frac{a_{\mathrm{ij}}L_{\mathrm{ij}}}{2}\right)}.$$

we then define the cumulative filtration on the segment CSGFR*_ij_* = *Q_i_* − Q_j_ as:

(s25) $${\mathrm{CSGFR}}_{\mathrm{ij}}=\frac{L_{\mathrm{ij}}}{R_{\mathrm{ij}}}\left(p_{i}+p_{j}‐{2p}_{\mathrm{BS}}\right)a_{\mathrm{ij}}\tanh\left(\frac{a_{\mathrm{ij}}L_{\mathrm{ij}}}{2}\right)=\frac{\frac{1}{L_{\mathrm{ij}}}\int_{\frac{{‐L}_{\mathrm{ij}}}{2}}^{\frac{L_{\mathrm{ij}}}{2}}\left(p_{\mathrm{ij}}\left(x\right)‐p_{\mathrm{BS}}\right)dx}{R_{\mathrm{ij}}^{f}},$$

As in Equation 1. If we let *J* be the set of nodes *j* connected to node *i*, conservation of blood flow at node *i* is represented by:

(26) $$\sum_{j\in J}Q_{i}=0,$$

which implies that

(27) $$\sum_{j\epsilon J}p_{i}\frac{{a_{\mathrm{ij}}L_{\mathrm{ij}}\tanh}^{2}\left(\frac{a_{\mathrm{ij}}L_{\mathrm{ij}}}{2}\right)+1}{R_{\mathrm{ij}}\tanh\left(\frac{a_{\mathrm{ij}}L_{\mathrm{ij}}}{2}\right)}+p_{j}\frac{{a_{\mathrm{ij}}L_{\mathrm{ij}}\tanh}^{2}\left(\frac{a_{\mathrm{ij}}L_{\mathrm{ij}}}{2}\right)‐1}{R_{\mathrm{ij}}\tanh\left(\frac{a_{\mathrm{ij}}L_{\mathrm{ij}}}{2}\right)}=\sum_{j\epsilon J}\frac{{{2a}_{\mathrm{ij}}L_{\mathrm{ij}}p}_{\mathrm{BS}}}{R_{\mathrm{ij}}}\tanh\left(\frac{a_{\mathrm{ij}}L_{\mathrm{ij}}}{2}\right)$$

Equation 27 allows for the solution of pressures at every node taking into account filtration along each segment.

### Erythrocyte distribution at network nodes

Incoming erythrocyte volume flow is summed at a network node and is then distributed according to a rule that seeks to capture the nonlinearity of blood phase separation at microvascular network bifurcations (Pries et al., 1990). For nodes *k* in *K* the set of nodes that flow into node *i*, and nodes *j* and l in *J* the set of two nodes that node *i* flows into, the erythrocyte volume *E_ij_* in capillary *ij* is:

(28) $$\begin{array}{c} E_{\mathrm{ij}}=\frac{\sum_{k\in K}E_{\mathrm{ik}}}{1+\exp\left(A_{\mathrm{ij}}‐B\;\text{logit}\left(\frac{FQ_{\mathrm{ij}}‐X_{0}}{1‐2X_{0}}\right)\right)}, \end{array}$$

where

$$\begin{array}{c} A_{\mathrm{ij}}=\frac{6.96\ln\left(\frac{D_{\mathrm{ij}}}{D_{\mathrm{il}}}\right)}{D_{f}} \end{array}$$

$$\begin{array}{c} B=1+\frac{6.98\left(1‐H_{f}\right)}{D_{f}} \end{array}$$

$$\begin{array}{c} X_{0}=\frac{0.4}{D_{f}} \end{array}$$

For *FQ_ij_* the fraction of blood flow distributed to node *j* and logit(*x*) = ln(*x*/(1 − *x*)). In calculating the parameters *A_ij_*, *B*, and *X*_0_, *D_f_* is the diameter of the parent branch. In the case that there is more than one parent branch, we combine the parent branches into one vessel with diameter such that the cross‐sectional area of the combined vessels is equal to the sum of the cross‐sectional areas of the actual parent branches. *H_f_* represents the discharge hematocrit present in the parent branch. This strategy is effective for estimating the blood flow distribution with two daughter branches but cannot predict this distribution for more than two daughter branches. In this case, we combine all but one of the daughter branches into a surrogate daughter branch with diameter such that the cross‐sectional area of the surrogate is equal to the sum of the combined daughter branch cross‐sectional areas. The uncombined daughter branch and the surrogate branch diameters are used with Equation 28 to calculate erythrocyte flow through the uncombined branch. Once flow through the uncombined branch is determined, this process is repeated with the branches that were combined to form the surrogate branch, until there are only two branches remaining.

### Apparent viscosity equations

Blood apparent viscosity used in calculating shear stress on the vessel walls and resistance of the capillaries in calculating pressures at network nodes is computed on capillary segment ij as follows:

(29) $$\begin{array}{c} \mu_{\mathrm{ij}}=\mu_{\mathrm{ij}}^{\mathrm{pl}}\lambda \left(D_{\mathrm{ij}},{\left(H_{t}\right)}_{\mathrm{ij}}\right), \end{array}$$

for *μ_pl_* the plasma viscosity in cP, which is a function of plasma protein concentration (Remuzzi et al., 1992):

(30) $$\begin{array}{c} \mu_{\mathrm{ij}}^{\mathrm{pl}}=0.274+0.177\frac{1}{L_{\mathrm{ij}}}\int_{0}^{L_{\mathrm{ij}}}C_{\mathrm{ij}}\left(x\right)dx. \end{array}$$

The non‐dimensional, experimentally derived scaling factor *λ* is based on vessel diameter *D* and discharge hematocrit *H_D_* (Pries et al., 1994), which is related in a nonlinear manner to the tube hematocrit *H_t_* and vessel diameter *D* due to the Faerheus–Lindquist effect:

(31) $$\begin{array}{c} {\left(H_{D}\right)}_{\mathrm{ij}}=\frac{{\left(H_{t}\right)}_{\mathrm{ij}}}{\frac{1}{2}\left(1+\exp\left(‐0.633\left(\frac{D_{\mathrm{ij}}}{D^{*}}‐1\right)\right)\right)}. \end{array}$$

In this equation *D** = 10.43 μm is defined as the minimum diameter vessel a red blood cell may pass through without altering its surface area (Ferrell et al., 2015). Equation 31 holds true for vessel diameters below 12 μm, however this relation breaks down for higher diameter vessels. As in (Ferrell et al., 2015), we assume a linear increase of *H_t_*/*H_D_* to unity at a diameter of 300 μm as a function of vessel diameter for vessels of diameter above 12 μm. While this is an oversimplification, only 8% of the capillary segments in the microvascular network have diameters over 12 μm and thus we believe this is a reasonable assumption. With this definition of *H_D_* (Pries et al., 1994),

(32) $$\begin{array}{c} \lambda \left(D_{\mathrm{ij}},{\left(H_{t}\right)}_{\mathrm{ij}}\right)=\frac{\left(1+\frac{\left({\left(\eta_{0.45}\right)}_{\mathrm{ij}}‐1\right)\left({\left(1‐{\left(H_{D}\right)}_{\mathrm{ij}}\right)}^{\gamma }‐1\right)}{{\left(1‐0.45\right)}^{\gamma }‐1}\frac{D_{\mathrm{ij}}^{2}}{{\left(D_{\mathrm{ij}}‐1.1\right)}^{2}}\right)D_{\mathrm{ij}}^{2}}{{\left(D_{\mathrm{ij}}‐1.1\right)}^{2}}, \end{array}$$

for

(33) $$\begin{array}{c} \gamma_{\mathrm{ij}}=\left(0.8+\exp\left(‐0.075D_{\mathrm{ij}}\right)\right)\left(‐1+\frac{1}{1+10^{‐11}D_{\mathrm{ij}}^{12}}\right)+\frac{1}{1+10^{‐11}D_{\mathrm{ij}}^{12}}. \end{array}$$

an experimentally derived shape parameter and

(34) $$\begin{array}{c} {\left(\eta_{0.45}\right)}_{\mathrm{ij}}=6\exp\left(‐0.085D_{\mathrm{ij}}\right)+3.2‐2.44\exp\left(‐0.06D_{\mathrm{ij}}^{0.645}\right) \end{array}.$$

The relative viscosity for a fixed ${\left(H_{D}\right)}_{\mathrm{ij}}$ of 0.45.

### Model algorithm

An iterative approach is taken to update the capillary filtration resistance $R_{\mathrm{ij}}^{f}$ such that the filtered volume is consistent with the colloid osmotic pressure which hinders filtration and is dependent upon plasma protein concentration. Apparent blood viscosity *μ_ij_* is similarly updated as erythrocyte flow and plasma protein concentration change with the volume filtered, which feeds back into nodal pressures and thus flows through each segment. We use Equations 8 and 12 to update $R_{\mathrm{ij}}^{f}$ and *μ_ij_*, respectively, however an additional step must be taken to control the change in these values to prevent oscillations. We propose a method based on control theory whereby the change in each $R_{\mathrm{ij}}^{f}$ and *μ_ij_* is dependent upon the maximum difference between these values at each iteration and the values obtained using Equations 8 and 12. Inlet and outlet pressure boundary conditions are set equal to MAP and peritubular capillary pressure, respectively, and inlet plasma protein concentration, hematocrit, and hydraulic conductivity (assumed the same for all capillary segments) are passed as parameters to the model. In describing each step of the algorithm, the exponent n is used to indicate the iteration number, beginning with *n* = 1 indicating the first iteration. The algorithm steps are as follows:

1. In the first iteration, initial values for $R_{\mathrm{ij}}^{f}$ are determined using Equation 8 on the basis that on average, the colloid osmotic pressure will be equal to 90% of the hydrostatic pressure gradient
  (35) $$\begin{array}{c} {\left(R_{\mathrm{ij}}^{f}\right)}^{n=1}=\frac{1}{0.1k\pi D_{\mathrm{ij}}L_{i}} \end{array}$$
2. Assuming zero erythrocytes in the network, and that apparent viscosity for all capillary segments is equal to a baseline plasma viscosity of 1.24 cP, Equation 11 is used to calculate the initial resistance of every capillary segment, $R_{\mathrm{ij}}^{n=}$
3. Equation 27 is used to calculate pressures at all nodes of the network.
4. Using Equations 21 and 22, the pressure and blood flow profiles $p_{\mathrm{ij}}^{n}\left(x\right)$ and $Q_{\mathrm{ij}}^{n}\left(x\right)$, respectively, are derived for each capillary segment.
5. CSGFR is calculated for each capillary segment using Equation 1. SNGFR is calculated by summing all CSGFRs.
6. Using Equation 28, erythrocyte volume is distributed throughout the network to calculate $E_{\mathrm{ij}}^{n}$ for each capillary segment.
7. Using Equations 7 and 10, the plasma protein concentration profile $C_{\mathrm{ij}}^{n}\left(x\right)$ is derived for each capillary segment of the network.
8. Equation 6 is used to calculate the colloid osmotic pressure profile $\Pi_{\mathrm{ij}}^{n}\left(x\right)$ on the length of each segment as a function of $C_{\mathrm{ij}}^{n}\left(x\right)$
9. Filtration resistances and apparent blood viscosities are updated for each capillary segment, first using Equations 8 and 12 to derive target $R_{\mathrm{ij}}^{f}$ and *μ_ij_*, denoted ($R_{\mathrm{ij}}^{f}$)^*^ and *μ_ij_* ^*^, respectively:
  (36) $${\left(R_{\mathrm{ij}}^{f}\right)}^{*}=\frac{\int_{0}^{L_{\mathrm{ij}}}\left(p_{\mathrm{ij}}^{n}\left(x\right)‐p_{\mathrm{BS}}\right)dx}{k\pi D_{\mathrm{ij}}L_{\mathrm{ij}}\int_{0}^{L_{\mathrm{ij}}}\left(p_{\mathrm{ij}}^{n}\left(x\right)‐p_{\mathrm{BS}}‐\Pi_{\mathrm{ij}}^{n}\left(x\right)\right)dx}.$$
  (37) $$\begin{array}{c} \mu_{\mathrm{ij}}^{*}=\mu_{\mathrm{pl}}^{n}\lambda \left(D_{\mathrm{ij}},{\left(H_{t}\right)}_{\mathrm{ij}}^{n}\right) \end{array}$$
  The hydrostatic pressure profile $p_{\mathrm{ij}}^{n}\left(x\right)$ is integrated analytically, whereas the colloid osmotic pressure profile $\Pi_{\mathrm{ij}}^{n}\left(x\right)$ is integrated by quadrature.
10. We then update the filtration resistances ($R_{\mathrm{ij}}^{f}$)*^n^* and apparent viscosities $\mu_{\mathrm{ij}}^{n}$ based on the difference between these values and the target values ($R_{\mathrm{ij}}^{f}$)^*^ and $\mu_{\mathrm{ij}}^{*}$. We define the term *α* to be the maximum relative difference of all ($R_{\mathrm{ij}}^{f}$)^n^ and $\mu_{\mathrm{ij}}^{n}$ with their respective target values
  (38) $$\begin{array}{c} \alpha =\max(\underset{\mathrm{ij}}{\beta \;\max}\frac{\left|\mu_{\mathrm{ij}}^{*}‐\mu_{\mathrm{ij}}^{n}\right|}{\mu_{\mathrm{ij}}^{n}},\underset{\mathrm{ij}}{\beta \;\max}\frac{\left|{\left(R_{\mathrm{ij}}^{f}\right)}^{*}‐{\left(R_{\mathrm{ij}}^{f}\right)}^{n}\right|}{{\left(R_{\mathrm{ij}}^{f}\right)}^{n}},1), \end{array}$$
  for *β* a smoothing parameter used to impose an upper bound on the amount that any given ($R_{\mathrm{ij}}^{f}$)*^n^* or $\mu_{\mathrm{ij}}^{n}$ may change from one iteration to the next. We then update ($R_{\mathrm{ij}}^{f}$)*^n^* and $\mu_{\mathrm{ij}}^{n}$ using *α*:
  (39) $$\begin{array}{c} \mu_{\mathrm{ij}}^{n+1}=\mu_{\mathrm{ij}}^{n}+\frac{\mu_{\mathrm{ij}}^{*}‐\mu_{\mathrm{ij}}^{n}}{\alpha }, \end{array}$$
  (40) $$\begin{array}{c} {\left(R_{\mathrm{ij}}^{f}\right)}^{n+1}={\left(R_{\mathrm{ij}}^{f}\right)}^{n}+\frac{{\left(R_{\mathrm{ij}}^{f}\right)}^{*}‐{\left(R_{\mathrm{ij}}^{f}\right)}^{n}}{\alpha },\;\text{if}\;{\left(R_{\mathrm{ij}}^{f}\right)}^{*}>0 \end{array},$$
  (41) $${\left(R_{\mathrm{ij}}^{f}\right)}^{n+1}=\left(1+\frac{1}{\beta }\right){\left(R_{\mathrm{ij}}^{f}\right)}^{n},\;\text{if}\;{\left(R_{\mathrm{ij}}^{f}\right)}^{*}<0$$
11. Steps 3–10 are repeated until both ($R_{\mathrm{ij}}^{f}$)*^n^* and $\mu_{\mathrm{ij}}^{n}$ converge, such that
  (42) $$\begin{array}{c} \max_{\mathrm{ij}}\frac{\left|\mu_{\mathrm{ij}}^{n+1}‐\mu_{\mathrm{ij}}^{n}\right|}{\mu_{\mathrm{ij}}^{n}}<\delta_{\mu }, \end{array}$$
  (43) $$\begin{array}{c} \max_{\mathrm{ij}}\frac{\left|{\left(R_{\mathrm{ij}}^{f}\right)}^{n+1}‐{\left(R_{\mathrm{ij}}^{f}\right)}^{n}\right|}{{\left(R_{\mathrm{ij}}^{f}\right)}^{n}}<\delta_{R}. \end{array}$$
  For *δ_μ_* = *δ_R_* = 1 × 10^–5^. The parameter *β* is used to control the degree to which the viscosities or the filtration resistances may change. In our simulations we use *β* = 3, such that the variables may change at most by 33.33%. This is similarly reflected in the case in which the target filtration resistance is negative, indicating that the colloid osmotic pressure exceeds the hydrostatic pressure gradient. In this case the corresponding filtration resistance is increased by a factor of 1/β, which will allow for the capillary to remain permeable unless it repeatedly has a colloid osmotic pressure that exceeds the hydrostatic pressure gradient. Thus, *β* may be chosen to determine the “smoothness” of the variables’ progression over the course of the iterations. For larger *β* there will be more iterations necessary, because more steps will be required to change the variables by the same amount. In the event that the maximum difference between the current iteration ($R_{\mathrm{ij}}^{f}$)*^n^* and $\mu_{\mathrm{ij}}^{n}$ and the calculated target values goes below 1/*β*, we say that the variables should then just converge to the target value, and the denominator in the derivative term of Equations 39 and 40 becomes 1.

In addition to the iterative procedure above, an iterative method is used to converge to the value of hydraulic conductivity k such that the model SNGFR matches that of a particular experimental study. The bisection method is used to try different values of *k* until both SNGFR and *k* converge. Two initial values of *k* are used to obtain an upper and lower bound on the SNGFR, and the average of the initial two *k* values is used to obtain either the next upper or lower bound, depending on if the target SNGFR is larger or smaller than the midpoint SNGFR estimate. Convergence of *k* and SNGFR are defined as

(44) $$\begin{array}{c} \frac{\left|k^{n+1}‐k^{n}\right|}{k^{n}}<\delta_{k}, \end{array}$$

(45) $$\begin{array}{c} \frac{\left|{\text{SNGFR}}^{n+1}‐{\text{SNGFR}}^{*}\right|}{{\text{SNGFR}}^{*}}<\delta_{S}. \end{array}$$

For SNGFR^*^ the target value for SNGFR based on micropuncture experiment data, and *δ_k_* = *δ_S_* = 1 × 10^–5^.

### Simulation parameters

Parameters used in the simulations not discussed in the text include afferent plasma protein C_A_, thickness of the glomerular basement membrane (GBM), Bowman's Space pressure *p_BS_*, and MAP, which are shown in Table 1 for each simulation condition. A peritubular capillary pressure of 15 mmHg, a minimum podocyte thickness $t_{\min}^{\text{pod}}$ of 5 nm, podocyte foot process height *h* ^pod^ of 0.3 μm, and a podocyte foot process width *w* ^pod^ of 0.17 μm are assumed for all simulation cases. Afferent and efferent arteriole resistances are included in Table 1. As in the sensitivity analysis, afferent and efferent resistances are calculated as in glomerular micropuncture experiments assuming a systemic hematocrit of 0.4 without taking into account the reduction of hematocrit seen in the microvasculature (Lipowsky et al., 1980).
